# Psychosocial distress and persistent adverse events in long‐term survivors of stage IV melanoma – a cross‐sectional questionnaire study

**DOI:** 10.1111/ddg.15712

**Published:** 2025-04-25

**Authors:** Markus Reitmajer, Norbert Schäffeler, Anne Bach, Lena Nanz, Teresa Amaral, Ulrike Leiter, Lukas Flatz, Andrea Forschner

**Affiliations:** ^1^ Department of Dermatology University Hospital Tuebingen Tübingen Germany; ^2^ Department of Psychosomatic Medicine and Psychotherapy University Hospital Tuebingen Tübingen Germany

**Keywords:** cancer survivors, long‐term survivors, Melanoma stage IV, psychosocial burden, quality of life, survivorship

## Abstract

**Background:**

Immune checkpoint inhibitors and targeted therapies have improved survival in patients with stage IV melanoma. However, the challenges faced by long‐term survivors remain unclear. The long‐term toxicity and psychosocial impact of these treatments in real‐world patients have yet to be reported.

**Material and Methods:**

We conducted a cross‐sectional questionnaire study using established screening tools, including the Hornheide Screening Instrument (HSI), the Distress Thermometer (DT) with the National Comprehensive Cancer Network (NCCN) problem list, and melanoma‐specific questions addressing persistent adverse events, social impairments, emotional needs, and financial concerns.

**Results:**

A total of 159 patients with stage IV melanoma (≥5 years after the initial diagnosis) were enrolled, of whom 93 completed the questionnaire. Approximately one‐third of DT/HSI values exceeded the threshold, indicating a need for psycho‐oncological support. More than 40% of patients reported persistent treatment‐related complaints. Financial and work‐related impacts were rare, affecting approximately 8% and 1% of patients, respectively.

**Conclusions:**

High rates of psychosocial distress and persistent adverse events were observed, highlighting the need for cancer survivorship programs in the follow‐up care of melanoma patients.

## INTRODUCTION

The era of immune checkpoint inhibition (ICI) and targeted therapy (TT) has significantly increased overall survival (OS) in stage IV melanoma patients.^1^, ^2^, ^3^, ^4^ Until 2010, only 5% of the patients with metastatic melanoma survived 5 years.^5^, ^6^, ^7^ Ten years later, the number increased to approximately 30%,^8^, ^9^, ^10^ and for patients with first‐line combined ICI (anti‐PD1/anti‐CTLA4) the 10‐year melanoma‐specific survival rate exceeds 50%.^11^, ^12^, ^13^ While OS remains the primary benchmark in cancer treatment, psychosocial aspects and coping mechanisms gain increasing importance as survival extends. The important question is, what price the long‐term survivors pay in terms of toxicity and psychosocial burden.^14^, ^15^

Limited knowledge exists about the psychosocial, physical, and financial burden of long‐term survivors with metastatic melanoma. Based on data from other cancer types, it can be expected that some of the long‐term survivors might live without any handicap, while others might experience severe impairments.^16^, ^17^ In other tumor entities survivorship programs have already been established and are now indispensable.^18^, ^19^, ^20^, ^21^ They address not only the fear of cancer recurrence, depression, and anxiety but also treatment‐specific adverse events and toxicity.^22^, ^23^, ^24^, ^25^, ^26^

In our previous study, we analyzed therapies, response rates, and tumor‐specific data in melanoma patients who survived more than 5 years after progressing to stage IV.^8^ This single‐center cross‐sectional questionnaire study aims to assess the specific circumstances of long‐term survivors.

## MATERIAL AND METHODS

### Study design

To identify patients diagnosed with stage IV melanoma between 01/01/2014 and 12/31/2017, we used the institutional database of the *Central Malignant Melanoma Registry* (CMMR). All 159 long‐term survivors were contacted via paper‐based questionnaires sent on 11/27/2023, with responses analyzed until 02/27/2024. A reminder was sent on 01/16/2024.

### Eligibility criteria and ethical approval

The following eligibility criteria had been applied: melanoma stage IV patients with OS after progressing to stage IV of at least 5 years and who were still alive at the cut‐off date 04/01/2023. All patients had been treated at the University Hospital Tuebingen. All patients provided written informed consent for data documentation in the melanoma registry for research and publication purposes. Ethical approval was granted by the University of Tübingen (reference number 466/2023BO2), and the study was conducted in accordance with the Declaration of Helsinki.

### Questionnaires

The questionnaire included two screening tools: the *Distress‐Thermometer* (DT) and *Hornheide‐Screening‐Instrument* (HSI),^15^, ^27^, ^28^, ^29^ along with self‐developed melanoma‐specific questions (MSQ) addressing therapy‐related complaints, such as adverse events from surgery, radiotherapy, and systemic therapy. It also covered work status, leisure activities, finances, and use of psychooncological support, social services, and cancer prevention programs (online supplementary Table S1). Additionally, we included a specific self‐assessment question (SA) to evaluate whether patients perceive a need for psycho‐oncological support.

Distress Thermometer scores range from 0 (no distress) to 10 (extreme distress), with values ≥ 5 considered above the threshold, potentially indicating a need for psychooncological support.^29^ The DT includes an attached NCCN problem list addressing practical, family, emotional, spiritual/religious, and physical concerns.^28^, ^29^, ^30^ The physical concern “dry mouth” was added to capture the commonly reported sicca symptom.

The HSI assesses seven areas: health condition, mental health, distress/burden besides the melanoma, availability of a person of trust, burdened family member, internal disturbance and the information status concerning disease and treatment. Scores ≥ 4 points suggest a need for psychosocial support.^15^, ^31^

### Statistical Analysis

Demographic and clinical data were analyzed descriptively. Correlations between HSI, DT, and SA were assessed using Spearman's rank correlation (two‐tailed). Binary logistic regression was conducted for each NCCN concern category and the MSQ to identify factors significantly associated with increased distress on the DT or the need for psychooncological support (cut‐off ≥ 5). Analyses were conducted with IBM^®^ SPSS^®^ Statistics 28 (IBM, Armonk, USA). Incomplete questionnaires were included using available data. Graphs were generated using GraphPad PRISM^®^ 9.5.0 (Dotmatics, Boston, USA).

## RESULTS

### Patient characteristics

A total of 93 out of 159 patients completed the questionnaire: 38 female (40.9%) and 55 males (59.1%). The median age of patients at the time of entering stage IV was 63 years, ranging from 25 to 83 years. The median time between stage IV entry and the last contact was 6 years, ranging from 5 to 9 years (Table 1).

**TABLE 1:** Patient characteristics.

Patient characteristics	Response to the questionnaire		Non‐responding patients	
	*n*	*% [range]*	*n*	*% [range]*
Number of patients	93	100	66	100
Sex				
Female	38	40.9	32	48.5
Male	55	59.1	34	51.5
Median years since diagnosis at time of the survey [range]	11	[6–36]	10	[6–33]
Age at the time of stage IV diagnosis median [range]	63	[25–83]	59	[30–85]
Years between entry into stage IV and last contact median [range]	6	[5–9]	6	[5–8]
Melanoma type				
Cutaneous	71	76.3	51	77.3
Unknown primary	9	9.7	8	12.1
Acral lentiginous	6	6.5	3	4.5
Uveal	5	5.4	3	4.5
Mucosal	2	2.2	1	1.5
Distant metastasis				
Lung	50	53.8	39	59.1
Liver	31	33.3	18	27.3
Central nervous system	22	23.7	15	22.3
BRAF^V600^ mutation				
Wildtype	48	51.6	32	48.5
Mutant	39	41.9	27	40.9
Unknown	6	6.5	7	10.6
Still receiving systemic therapy at the time of the survey (04/01/2023)	14	15.1	7	10.6
ICI at any time point				
Yes	72	77.4	42	63.6
No	21	22.6	24	36.4
TT at any time point				
Yes	22	23.7	14	21.2
No	71	76.3	52	78.8
Palliative systemic therapies				
One	80	86.0	51	77.3
Two	36	38.7	28	42.4
Three or more	25	26.9	19	28.8
Without any palliative therapy (28 patients)	13	14.0	15	22.7
Adjuvant systemic therapy only	6	6.5	5	7.6
Adjuvant radiotherapy only	2	2.2	2	3.0
Surgery/stereotaxy/ radiofrequency ablation only	5	5.4	8	12.1
Radiotherapy at any time point				
Yes	39	41.9	23	34.8
No	54	58.1	43	65.2
Radiotherapy				
Brain	12	12.9	9	13.6
Soft tissue/regional lymph nodes	21	22.6	11	16.7

*Abbr*.: ICI, immune checkpoint inhibition, TT, targeted therapy

In the group of patients without response, nine questionnaires (5.7%) could not be delivered due to unknown address changes. An overview of the characteristics of all contacted patients is presented in Table 1.

### Strong correlation between HSI and DT

A two‐tailed Spearman correlation analysis was conducted to evaluate potential associations between HSI, DT, and SA. A strong correlation was observed between HSI and DT (ρ = 0.674, n = 87), while moderate correlations were found between HSI and SA (ρ = 0.457, n = 90) and between DT and SA (ρ = 0.351, n = 87). Both HSI (n = 29/90, 32.2%) and DT (n = 32/88, 36.4%) identified a similar proportion of patients in need of psychooncological support. Notably, the percentage of patients indicating a need for psycho‐oncological support in the SA (n = 11/90, 12.2%) was lower than that identified by professional assessment instruments (HSI, DT) (online supplementary Table 2). More than one‐third of long‐term melanoma patients reported DT values above the threshold (36.4%, n = 32/88).

### Physical concerns were the most common issues on the NCCN problem list

**FIGURE 1:**
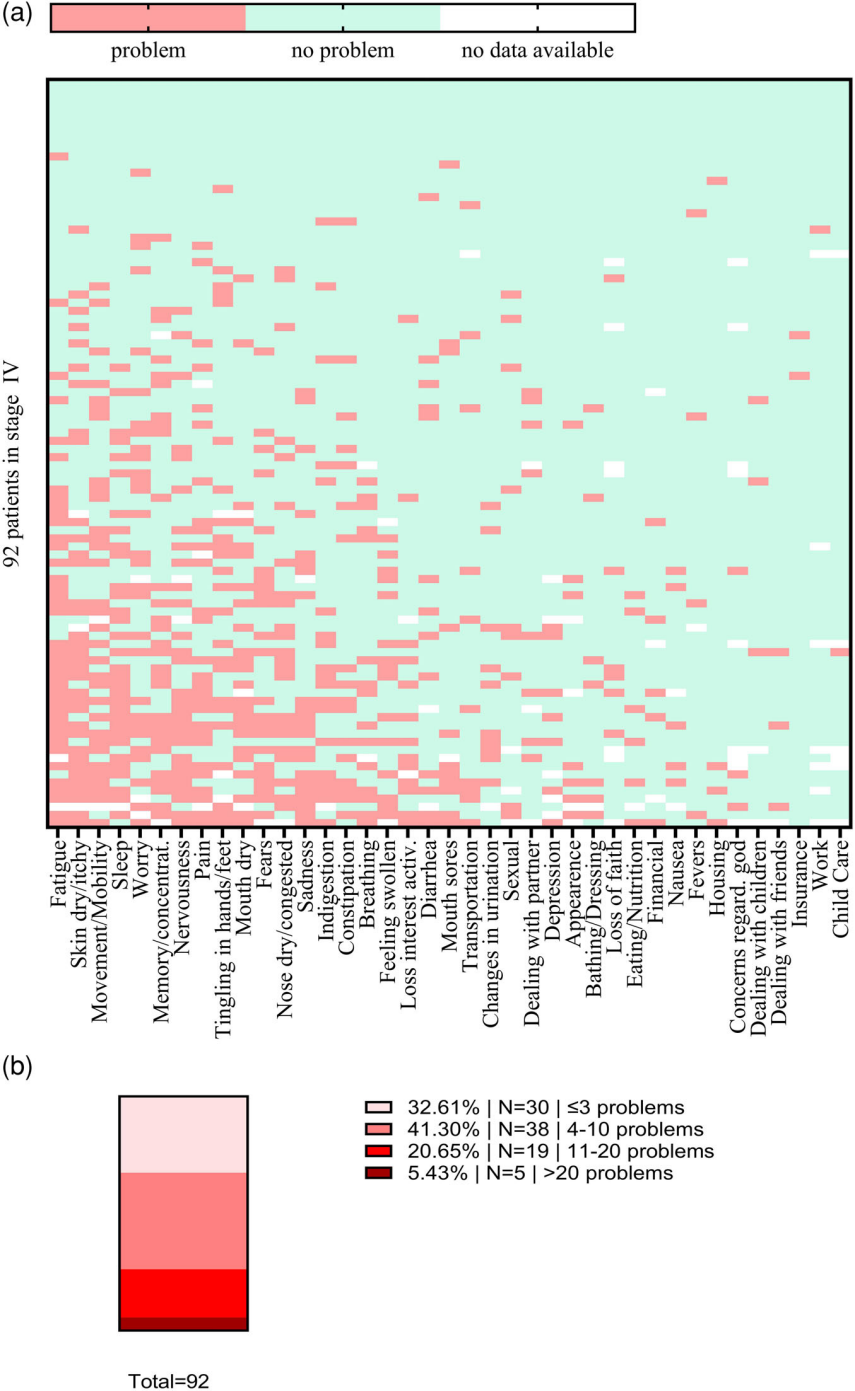
Results of the NCCN questionnaire. (a) The heat map provides a visual overview of all issues identified by the all 92 stage IV patients who completed the NCCN questionnaire. Horizontal Axis: The most frequent problems are arranged along the left side of the heat map. Vertical Axis: Patients experiencing the most problems are located towards the bottom of the heat map. (b) Patients were categorized into four groups based on the NCCN Problem List: Group 1 reported ≤ 3 problems on the NCCN Problem List, Group 2 reported 4–10 problems, Group 3 reported 11–19 problems, and Group 4 reported ≥ 20 problems.

Ninety‐two out of 93 stage IV melanoma patients completed the NCCN problem list at least partially. Fatigue was the most common issue in stage IV (43.3%, n = 39/90) (Figure 1a). Physical concerns were the most frequently mentioned complaint group, while “family,” “practical,” and “spiritual/religious” concerns only rarely were mentioned (Table 2). Concerning the total number of problems mentioned, approximately one third of the patients (32.6%, n = 30) reported in total ≤ 3 problems, 41.3% (n = 38) reported in total 4–10 problems, and 20.7% (n = 19) ≥ 11 problems. We also identified a small subgroup of 5.4% (n = 5) who reported more than 20 issues (Figure 1b). Approximately one‐fifth of the patients had at least one contact with the psycho‐oncological or social service (Table 3).

**TABLE 2:** NCCN‐problem list ranked from most frequent to least frequent.

NNCN problem list	Melanoma AJCC Stage IV		Category of concern
	*Positive/total*	*%*	
Fatigue	39/90	43.3	Physical
Dry/itchy skin	35/89	39.3	Physical
Movement/Mobility	32/90	35.6	Physical
Sleep	32/91	35.2	Physical
Worry	29/89	32.6	Emotional
Memory or concentration	29/90	32.2	Physical
Nervousness	29/90	32.2	Emotional
Pain	28/89	31.5	Physical
Paresthesia/tingling in hands/feet	24/89	27.0	Physical
Dry mouth	24/89	27.0	Physical
Fears	23/91	25.3	Emotional
Nose dry/congested	23/91	25.3	Physical
Sadness	22/91	24.2	Emotional
Indigestion	19/91	20.9	Physical
Constipation	17/92	18.5	Physical
Breathing	15/90	16.7	Physical
Feeling swollen/edema	15/90	16.7	Physical
Loss of interest in everyday activities	13/90	14.4	Emotional
Diarrhea	13/90	14.4	Physical
Mouth sores	11/91	12.1	Physical
Transportation	10/91	11.0	Practical
Changes in urination	10/91	11.0	Physical
Sexual problems	9/89	10.1	Physical
Dealing with Partner	8/89	9.0	Family
Depression	7/87	8.0	Emotional
Physical appearance	7/88	8.0	Physical
Bathing/Dressing	7/91	7.7	Physical
Loss of faith	6/85	7.1	Spiritual/Religious
Eating/Nutrition	6/91	6.6	Physical
Financial situation	5/89	5.6	Practical
Nausea	5/90	5.6	Physical
Fevers	4/91	4.4	Physical
Housing situation	4/92	4.3	Practical
Concerns regarding god	3/83	3.6	Spiritual/Religious
Dealing with children	3/90	3.3	Family
Dealing with friends	3/91	3.3	Family
Insurance	2/92	2.2	Practical
Work/school	1/85	1.2	Practical
Childcare	1/86	1.2	Practical

To give details about each NCCN concern group, we provided a color‐coding system: practical = yellow, emotional = orange, family = green, spiritual = blue, physical = red.

**TABLE 3:** Evaluation of the melanoma‐specific questions (MSQ) in the questionnaire.

Melanoma‐specific questions	Positive/total	Percent	Median time since last therapy in years [range]
*Still experiencing issues due to a systemic drug therapy*			
Yes	37/80	46.3	6 [0–11]
No	43/80	53.8	6 [0–9]
N/a	0/80	0	
*Still experiencing issues due to surgery*			
Yes	39/93	41.9	11 [7–29]
No	50/93	53.8	11 [7–37]
N/a	4/93	4.3	12 [11–16]
*Still experiencing issues due to radiotherapy*			
Yes	17/39	43.6	8 [2–13]
No	22/39	56.4	8 [3–14]
N/a	0/39	0	
*Experiencing financial limitations due to their melanoma stage IV diagnosis*			
Yes	7/93	7.5	
No	81/93	87.1	
N/a	5/93	5.4	
*Experiencing limitations in your leisure time due to your melanoma disease*			
Yes	30/93	32.3	
No	57/93	61.3	
N/a	6/93	6.5	
*Feel affected by their melanoma diagnosis at work*			
Yes	1/93	1.1	
No	29/93	31.2	
Not working/retired	60/93	64.5	
N/a	3/93	3.2	
*Support from the psycho‐oncological service*			
Yes	21/93	22.6	
No	61/93	65.6	
N/a	11/93	11.8	
*Support from social counseling*			
Yes	19/93	20.4	
No	65/93	70.0	
N/a	9/93	9.7	
*Regularly attend other cancer screening examinations (colorectal, breast, or prostate cancer)*			
Yes	73/93	78.5	
No	17/93	18.3	
N/a	3/93	3.2	
*Feeling adequately informed about the risks and benefits of systemic therapy for melanoma*			
Yes	76/93	81.7	
No	15/93	16.1	
N/a	2/93	2.2	

*Abbr*.: n/a, not answered; n, number of patients

### Financial limitations and impact on work due to melanoma stage IV

Approximately 8% (n = 7/93) of the patients reported financial limitations contributed to the melanoma stage IV diagnosis. Only one patient felt limited at work, while most of the patients (64.5%, n = 60/93) were not working. About one third of the patients (31.2%, n = 29/93) did not feel like being affected at work by their melanoma diagnosis. Of note, the survey did not cover whether the patients had stopped working due to melanoma. Approximately one‐third (32.3%, n = 30/93) felt limited in their leisure time due to melanoma disease (Table 3). The most common reason in detail was the limitation in physical mobility (n = 10), impairing patients during leisure time. Neither financial nor work limitations were significantly associated with DT values ≥ 5.

### Over forty percent of stage IV melanoma patients report persistent complaints due to treatment in the past

**FIGURE 2:**
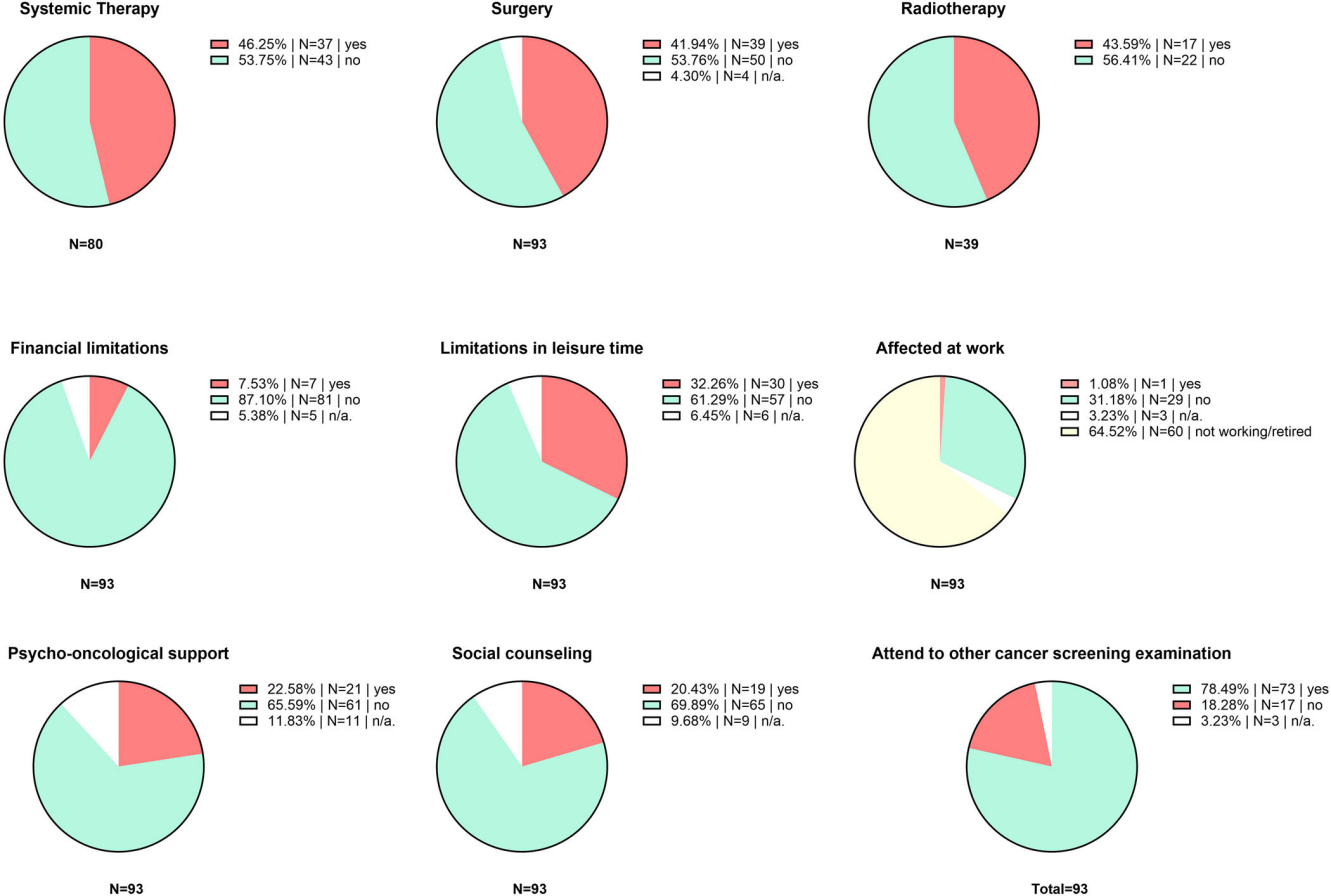
Results of the MSQ (melanoma specific questions) of the survey. The Figure shows the currently present adverse events and issues related to systemic drug therapy, surgery, and radiotherapy. *Abbr*.: n/a, not answered, N, number of patients

Forty‐six percent (n = 37/80) of patients who received at least one systemic therapy reported persisting physical issues that they attribute to former systemic drug therapy (Figure 2, Table 3). Among these, skin issues (n = 23), dry mouth (n = 15), and joint discomfort (n = 15) were the most frequently mentioned problems. The median time since ending the last therapy was 6 years, ranging from 0 to 11 years. Thirty‐three of these patients (89.2%) had at least one cycle of ICI, and nine patients (18.9%) received at least one cycle of targeted therapy (TT). A similarly high number (43.6%, n = 17/39) of patients reported persistent symptoms due to radiation therapy in the past (Figure 2, Table 3). The most commonly mentioned issues were restrictions in physical ability (n = 7), edema/swelling (n = 5), and numbness of the skin (n = 5). The median time since the last radiotherapy was 8 years, ranging from 2 to 23 years.

Among long‐term survivors in stage IV, 41.9% (n = 39/93) still suffer from physical discomfort that they attribute to past surgical procedures (Figure 2, Table 3). Additionally, 41.8% (n = 5/12) of patients who received cerebral radiotherapy report issues with memory and concentration problems. The median time since the last surgical procedure was 11 years, ranging from 7 to 29 years. Twenty‐two patients currently report numbness of the skin, 17 patients report restricted movement, and 15 patients still report edema due to past surgical procedures.

However, 81.7% (n = 76/93) of patients report that they feel well informed about the potential risks and benefits of systemic therapies. Additionally, 78.5% (n = 73/93) of patients reported that they regularly attend other cancer screening examinations, such as colorectal, breast, or prostate cancer screenings (Table 3).

### Factors that are significantly associated with DT values ≥ 5

Binary logistic regression for each NCCN problem category and the MSQ identified nine factors that were significantly associated with DT values ≥ 5, indicating a need for psycho‐oncological support (Table 4). Six factors from the NCCN problem list were significant: transportation, fears, nervousness, sleep, exercise/movement, and dry/itchy skin. Two MSQ factors were also significant: ongoing issues from systemic therapy and limitations in leisure activities due to melanoma. Additionally, patients' self‐assessment of needing psycho‐oncological help was significant. Non‐significant factors are detailed in online supplementary Table S3.

**TABLE 4:** Significant factors associated with an increased burden in DT (cut‐off ≥ 5).

Factors	Category	n	B	SE	p	Odds ratio [Exp(B)]	95% CI Exp(B)		Cox‐/Snell R^2^	Nagelkerke's R^2^
							*Lower bound*	*Upper bound*		
Exercise/movement	NCCN	73	5.262	2.097	0.012	192.953	3.167	11754.628	0.522	0.733
Sleep	NCCN	73	4.283	1.691	0.011	72.485	2.636	1993.261	0.522	0.733
Dry/itchy Skin	NCCN	73	3.645	1.526	0.017	38.281	1.922	762.451	0.522	0.733
Fears	NCCN	83	2.393	0.870	0.006	10.943	1.989	60.216	0.368	0.507
Transportation	NCCN	78	2.381	1.141	0.037	10.812	1.156	101.137	0.168	0.233
Self assessment (in need of psychooncological help)	MSQ	87	1.754	0.721	0.015	5.778	1.407	23.720	0.075	0.102
Nervousness	NCCN	83	1.558	0.688	0.024	4.749	1.232	18.302	0.368	0.507
Still experiencing issues due to a systemic therapy	MSQ	80	1.407	0.521	0.007	4.082	1.469	11.343	0.142	0.193
Experiencing limitations in your leisure time due to your melanoma disease	MSQ	83	1.218	0.489	0.013	3.379	1.295	8.816	0.074	0.102

*Abbr*.: NCCN, National Comprehensive Cancer Network problem list; MSQ, melanoma specific questions; n, number of patients; B, regression coefficient B; SE, standard error; Exp(B), exponentiation of B; CI, confidence interval

## DISCUSSION

Long‐term cancer survivors are often considered cured; however, it remains uncertain whether they can truly be regarded as healthy and fully resilient compared to the general population. In other cancer entities, persistent therapy adverse events and psychosocial impairments have been demonstrated in long‐term survivors.^21^, ^32^, ^33^, ^34^, ^35^, ^36^ To our knowledge, this is the first survey of long‐term melanoma survivors who have survived at least 5 years after entering stage IV, focusing on psychooncological assessment, financial burdens, and potential long‐term adverse events with established screening instruments (DT, HSI) and individual questions adapted on frequently applied melanoma therapies considering therapy‐associated adverse effects.

In a recent study of long‐term survivors with ICI in unresectable stages III and IV who survived more than 12 months, patients reported fatigue (28%), aching joints (17%) and aching muscles (12%) as the most frequent symptoms experienced.^37^ Similarly, in our cohort, fatigue (43%) and movement/mobility issues (36%) are the most common issues. Additionally, we identified itchy/dry skin (39%) as the second most frequently reported problem in the NCCN problem list. Itchy/dry skin was also identified as a significant factor related to DT values ≥ 5. We also asked patients about any organs that were persistently affected due to previous systemic drug therapy. The most frequently mentioned organ by patients was the skin. One reason could be that cutaneous immune‐mediated effects might be related to improved outcome with ICI and therefore are more frequent in this cohort of long‐term survivors. There are several hints in the literature describing that the occurrence of cutaneous immune‐related adverse effects (irAEs; especially vitiligo) might be associated with improved outcome with ICI.^38^, ^39^, ^40^ While only a small proportion of patients (15%) are still receiving systemic therapy at the time of the survey, it has to be assumed that the irAEs in most of the patients persist even after the treatment has ended.

In our survey, over 40% of stage IV melanoma patients reported ongoing problems due to prior treatments, aligning with a previous study that found a 43% rate of chronic irAEs in patients with stage III–IV melanoma treated with anti‐PD‐1.^41^ We observed that these persistent adverse events were significantly associated with a higher proportion of patients with psychooncological distress values ≥ 5. Almost 90% of all patients received at least one course of ICI, while only around 20% had at least one course of TT. This is consistent with observations in the adjuvant setting that TT causes higher transient but lower persistent toxicity compared to ICI. In this retrospective analysis, persistent irAEs were detected in 3% (95% confidence interval [CI]: 1.6–5.3%) of the TT group and 26.3% (95% CI: 20.5–32.9%) of the ICI group.^42^ Of note, we found no significant association between DT ≥ 5 and persistent adverse events continuing after radiation therapy or surgery. Due to the small sample size (n = 80) and wide confidence intervals in some subgroups, our estimates may lack precision. Larger multicenter studies are necessary to draw definitive conclusions.

While the socioeconomic consequences in long‐term survivors of other cancer entities, such as unemployment, early retirement, and resulting financial burdens, are well documented, the relevance of this topic remains unclear in the group of long‐term melanoma stage IV survivors.^32^, ^43^ However, we could show that no complaint within the group of practical concerns was ranked within the top ten of the most frequently reported complaints. Only 8% of the patients reported financial limitations. Notably, these results exclusively cover skin cancer centers within the DeCOG, where legally regulated social protection mechanisms regarding finances and health insurance are well established. Thus, practical and social concerns may be more relevant in countries with different health‐care and social‐care systems. Apart from this, the age at the time of entering stage IV melanoma in our cohort was close to the normal retirement age in Germany. This is also reflected in the very small percentage (1%) of patients who reported feeling limited at work due to their melanoma. 67% of our patients are no longer working. However, we did not assess whether these patients retired due to reaching regular pension age or earlier due to melanoma or other reasons.

When comparing the Distress Thermometer values of our long‐term stage IV melanoma patients with a published cohort of stage I patients (not solely long‐term survivors), we found that long‐term survivors in stage IV actually exhibit a lower percentage of threshold exceedance.^44^ One could suppose that successful adaptation and illness coping processes might be widespread among long‐term survivors. On the other side, effective coping strategies might also have contributed to long‐term survival in stage IV.^45^ This aligns with the fact that nearly 80% of patients reported regularly attending other cancer screening examinations, indicating a specific form of intentional health behavior.

High rates of psychosocial distress and persistent adverse events were identified in this group of long‐term melanoma survivors who have lived at least 5 years after progressing to stage IV. However, a direct comparison with individuals unaffected by melanoma was not included in this cross‐sectional questionnaire study. Therefore, we cannot conclusively confirm that the issues reported by the patients were caused solely by melanoma or other potential influencing factors.

Our data suggest that stage IV melanoma survivors should be considered differently from long‐term survivors of other cancer types. Financial issues and social impairments seem to be subordinate, while physical complaints are frequent. High rates of persistent adverse events were identified, underscoring the importance of implementing cancer survivorship programs in the follow‐up care of melanoma patients. Further multicenter studies worldwide are necessary to fully understand this heterogeneous and growing patient group.

## CONFLICT OF INTEREST STATEMENT

M.R. received funding as part of the Junior Clinician Scientists Program of the University of Tübingen (application no. 523‐0‐0) and travel support from Almirall Hermal and Pierre Fabre, outside the submitted work. T.A. reports personal honoraria from BMS, CeCaVa, Novartis, Philogen, Pierre Fabre, and Delcath; institutional financial support from iFIT, Neracare, Novartis, Sanofi, and SkylineDX; and an institutional research grant from Novartis. He is chair of the ESMO Leadership Development Committee and has received travel support from Neracare. Additionally, he reports fees for conducting clinical studies, with payments to the institution, from Agenus, AstraZeneca, BioNTech, BMS, HUYA, iFIT, Immunocore, IO Biotech, MNI, MSD, Neracare, Novartis, Pascoe, Pfizer, Philogen, Regeneron, Roche, Sanofi, SkylineDX, and Unicancer, all outside the submitted work. U.L. received payments/honoraria for lectures and presentations from Sun Pharma and Sanofi, travel support from Sun Pharma, Sanofi, and Pierre Fabre, and serves as a board member for Pierre Fabre, Sun Pharma, Sanofi, MSD, Novartis, DECOG, SCOPE, and EADO, all outside the submitted work. L.F. received grants from Hookipa Pharma, the Swiss Cancer League, the German Research Foundation, Immunophotonics, and Mundipharma. L.F. also received consulting fees from Philogen and support for attending meetings or travel from Philogen and Hookipa Pharma. Furthermore, L.F. serves on the board for the University of Basel (TIL trial, unpaid) and is the founder of Hookipa Pharma, Schmelzberg, Humion, and Abtherix, all outside the submitted work. A.F. served as a consultant for Novartis, MSD, BMS, Pierre Fabre, and Immunocore; received travel support from Novartis, BMS, and Pierre Fabre; received speaker fees from Novartis, Delcath, BMS, and MSD; and reports institutional research grants from the BMS Stiftung Immunonkologie, all outside the submitted work. L.N., A.B., and N.S. declare no conflicts of interest.

## Supporting information



Supplementary information

Supplementary information

Supplementary information
