# Supplementary material for: Psychosocial distress and persistent adverse events in long‐term survivors of stage IV melanoma – a cross‐sectional questionnaire study
Source: J Dtsch Dermatol Ges. 2025 Apr 25;23(7):832–42. doi: 10.1111/ddg.15712 (PMC12257058; doi:10.1111/ddg.15712)
Supplement: Supplementary file 1 — Supplementary information [file DDG-23-832-s003.docx]

| How have you been feeling physically over the past three days? | | | | | | | | Quite good  ❑ 0 | Average  ❑ 1 | | Rather bad ❑ 2 |  |  |
| --- | --- | --- | --- | --- | --- | --- | --- | --- | --- | --- | --- | --- | --- |
| How have you been feeling emotionally over the past three days? | | | | | | | | Quite good  ❑ 0 | Average  ❑ 1 | | Rather bad ❑ 2 |  |  |
| Is there something that is causing you significant stress, unrelated to your melanoma diagnosis? | | | | | | | | Yes  ❑ 2 | No  ❑ 0 | |  |  |  |
| Do you have someone you can talk to about your worries and fears? | | | | | | | | Yes  ❑ 0 | No  ❑ 2 | |  |  |  |
| Is there someone in your family who is particularly burdened by your melanoma diagnosis? | | | | | | | | Yes  ❑ 2 | No  ❑ 0 | |  |  |  |
| Are you able to find inner calm during the day? | | | | | | | | Yes  ❑ 0 | No  ❑ 2 | |  |  |  |
| How well do you feel informed about your illness and treatment? | | | | | | | | Quite good  ❑ 0 | Average  ❑ 1 | | Rather bad ❑ 2 |  |  |
| Self-assessment of your current personal situation:  Do you currently need support in coping with your illness or a psycho-oncological treatment? | | | | | | | | Yes  ❑ | No  ❑ | |  |  |  |
| Please circle the number on the thermometer to the right (0-10) that best describes how distressed you have felt over the past week, including today: 10=Extremely distressed. 0=Not distressed at all. | | | | | | | | 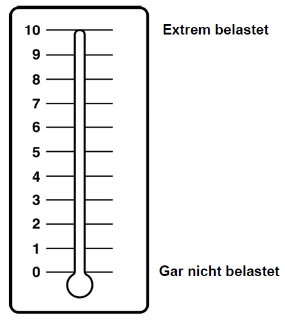 | | | |  |  |
| Please specify whether you have had problems in any of the following areas over the past week, including today. For each area, mark YES or NO. | | | | | | | | | | | |  |  |
| YES | NO | | **Practical concerns** | YES | | NO | **Physical concerns** | | | | | |  |
| □ | □ | | Housing situation | □ | | □ | Pain | | | | | |  |
| □ | □ | | Insurance | □ | | □ | Nausea | | | | | |  |
| □ | □ | | Work/school | □ | | □ | Fatigue | | | | | |  |
| □ | □ | | Transportation | □ | | □ | Sleep | | | | | |  |
| □ | □ | | Child care | □ | | □ | Exercise/Movement | | | | | |  |
| □ | □ | | Financial situation | □ | | □ | Bathing/dressing | | | | | |  |
| YES | NO | | **Familiy concerns** | □ | | □ | Physical appearance | | | | | |  |
| □ | □ | | Dealing with partner | □ | | □ | Breathing | | | | | |  |
| □ | □ | | Dealing with children | □ | | □ | Mouth sores | | | | | |  |
| □ | □ | | Dealing with friends | □ | | □ | Mouth dry | | | | | |  |
| YES | NO | | **Emotional concerns** | □ | | □ | Eating/Nutrition | | | | | |  |
| □ | □ | | Worry | □ | | □ | Indigestion | | | | | |  |
| □ | □ | | Fears | □ | | □ | Constipation | | | | | |  |
| □ | □ | | Sadness | □ | | □ | Diarrhea | | | | | |  |
| □ | □ | | Depression | □ | | □ | Changes in urination | | | | | |  |
| □ | □ | | Nervousness | □ | | □ | Fevers | | | | | |  |
| □ | □ | | Loss of interest | □ | | □ | Dry/itchy skin | | | | | |  |
|  |  | | in everyday activities | □ | | □ | Nose dry/congested | | | | | |  |
|  |  | |  | □ | | □ | Tingling in hands/feet | | | | | |  |
| YES | NO | | **Spiritual/religious concerns** | □ | | □ | Feeling swollen/ | | | | | |  |
| □ | □ | | Concerns regading god |  | |  | edema | | | | | |  |
| □ | □ | | Loss of faith | □ | | □ | Memory/Concentration | | | | | |  |
|  |  | |  | □ | | □ | Sexual problems | | | | | |  |
| Are you currently experiencing any side effects from a previous systemic drug therapy for melanoma? | | | | | | | | | | | □ Yes  □ No | | |
|  | | | If **yes**: In which area are you experiencing problems? | | | | | | | |  | | |
|  | | | □ Lung | | | □ Skin | | | | |  | | |
|  | | | □ Pituitary | | | □ Heart | | | | |  | | |
|  | | | □ Dry mouth | | | □ Joints | | | | |  | | |
|  | | | □ Mental health | | | □ Sexuality | | | | |  | | |
|  | | | □ Other: | | | | | | | |  | | |
| Do you feel adequately informed about the risks and benefits of medication therapy for melanoma? | | | | | | | | | | | □ Yes  □ No | | |
|  | | | If **no**: What aspects do you feel were not adequately addressed? | | | | | | | |  | | |
|  | | | □ Long-term side effects | | | □ Therapy failure | | | | |  | | |
|  | | | □ Therapy success rate | | | □ Commitment (e.g. time) | | | | |  | | |
| Do you currently have any complaints due to a previous surgical therapy for melanoma? | | | | | | | | | | | □ Yes  □ No | | |
|  | | | If **yes**: What problems are you experiencing? | | | | | | | |  | | |
|  | | | □ Hardening of the skin | | | □ Pain | | | | |  | | |
|  | | | □ Numbness in the skin | | | □ Edema/swelling tendency | | | | |  | | |
|  | | | □ Limitation of movement | | | □ Other: | | | | |  | | |
| Do you currently have any complaints due to a previous radiation therapy for melanoma? | | | | | | | | | | | □ Yes  □ No | | |
|  | | | If **yes**: What problems are you experiencing? | | | | | | | |  | | |
|  | | | □ Hardening of the skin | | | □ Pain | | | | |  | | |
|  | | | □ Numbness in the skin | | | □ Edema/swelling tendency | | | | |  | | |
|  | | | □ Limitation of movement | | | □ Other: | | | | |  | | |
| Do you attend cancer screenings (e.g., for colon, breast, or prostate cancer)? | | | | | | | | | | | □ Yes  □ No | | |
| Do you feel financially constrained by your melanoma diagnosis? | | | | | | | | | | | □ Yes  □ No | | |
|  | | | If **yes**: why? | | | | | | | |  | | |
| Do you feel limited in your workplace due to your melanoma diagnosis? | | | | | | | | | | | □ Yes  □ No  □ Unemployed | | |
|  | | | If **yes**: why? | | | | | | | |  | | |
| Do you feel limited in your leisure time due to your melanoma diagnosis? | | | | | | | | | | | □ Yes  □ No | | |
|  | | | If **yes**: why? | | | | | | | |  | | |
| Have you received or are you currently receiving support from the psycho-oncological service or other psychologists? | | | | | | | | | | | □ Yes  □ No | | |
| Have you received or are you currently receiving support from social counseling? | | | | | | | | | | | □ Yes  □ No | | |
| Is there anything else you would like to share with us? Do you have any suggestions for improvement? | | | | | | | | | | | | | |

**Online supplementary table 1 Survey for identifying burden in cancer diseases**

The questionnaire was sent to the patients in German and was translated into English for publication purposes.
