# Supplementary material for: Psychosocial distress and persistent adverse events in long‐term survivors of stage IV melanoma – a cross‐sectional questionnaire study
Source: J Dtsch Dermatol Ges. 2025 Apr 25;23(7):832–42. doi: 10.1111/ddg.15712 (PMC12257058; doi:10.1111/ddg.15712)
Supplement: Supplementary file 3 — Supplementary information [file DDG-23-832-s001.docx]

| **Factors** | **Category** | **N** | **B** | **SE** | **p** | **Odds ratio**  **[Exp(B)]** | **95% CI Exp(B)** | | **Cox-/Snell R Quadrat** | **Nagelkerkes R-Quadrat** |
| --- | --- | --- | --- | --- | --- | --- | --- | --- | --- | --- |
|  |  |  |  |  |  |  | **lower bound** | **upper bound** |  |  |
| **Gender** | MSQ | 88 | 0.666 | 1.947 | 0.141 | 1.947 | 0.802 | 4.726 | 0.024 | 0.034 |
| **Still experiencing issues due to surgery** | MSQ | 80 | 0.579 | 0.562 | 0.303 | 1.785 | 0.593 | 5.371 | 0.142 | 0.193 |
| **Still experiencing issues due to radiotherapy** | MSQ | 80 | 0.055 | 0.651 | 0.933 | 1.057 | 0.295 | 3.784 | 0.142 | 0.193 |
| **Support from the psycho-oncological service** | MSQ | 70 | 0.757 | 0.614 | 0.218 | 2.131 | 0.640 | 7.102 | 0.025 | 0.035 |
| **Support from social counseling** | MSQ | 70 | 0.063 | 0.615 | 0.918 | 1.065 | 0.319 | 3.558 | 0.025 | 0.035 |
| **Best response under 1. ST (CR, PR, SD vs. PD)** | MSQ | 75 | 0.405 | 0.527 | 0.442 | 1.500 | 0.534 | 4.214 | 0.008 | 0.011 |
| **Best response under 2. ST (CR, PR, SD vs. PD)** | MSQ | 32 | -1.163 | 1.183 | 0.326 | 0.313 | 0.031 | 3.177 | 0.035 | 0.047 |
| **Still undergoing systemic therapy** | MSQ | 88 | -0.616 | 0.707 | 0.384 | 0.540 | 0.135 | 2.161 | 0.009 | 0.013 |
| **Regularly attend other cancer screening examinations (colorectal, breast, or prostate cancer)** | MSQ | 85 | -1.466 | 0.798 | 0.066 | 0.231 | 0.048 | 1.102 | 0.050 | 0.069 |
| **Experiencing financial limitations due to their melanoma stage IV diagnosis** | MSQ | 83 | 1.570 | 0.871 | 0.071 | 4.808 | 0.872 | 26.500 | 0.043 | 0.059 |
| **Housing situation** | NCCN | 78 | -20.261 | 40192.970 | 1.000 | 0.001 | 0.001 | - | 0.168 | 0.233 |
| **Insurance** | NCCN | 78 | -21.845 | 25818.486 | 0.999 | 0.001 | 0.001 | - | 0.168 | 0.233 |
| **Work/school** | NCCN | 78 | -20.261 | 40192.970 | 1.000 | 0.001 | 0.001 | - | 0.168 | 0.233 |
| **Child care** | NCCN | 78 | 22.144 | 40192.969 | 1.000 | 4141744977,929 | 0.001 | - | 0.168 | 0.233 |
| **Financial situation** | NCCN | 78 | 1.698 | 1.232 | 0.168 | 5.462 | 0.488 | 61.098 | 0.168 | 0.233 |
| **Dealing with partner** | NCCN | 84 | 0.365 | 0.803 | 0.650 | 1..440 | 0.299 | 0.6943 | 0.070 | 0.960 |
| **Dealing with children** | NCCN | 84 | -20,196 | 21546,627 | 0.999 | 0.001 | 0.001 | - | 0.070 | 0.960 |
| **Dealing with friends** | NCCN | 84 | 41,122 | 33200,162 | 0.999 | 7.227E+17 | 0.001 | - | 0.070 | 0.960 |
| **Worry** | NCCN | 83 | -0.095 | 0.788 | 0.904 | 0.909 | 0.194 | 4.261 | 0.368 | 0.507 |
| **Sadness** | NCCN | 83 | 2.393 | 0.870 | 0.263 | 2.550 | 0.495 | 13.133 | 0.368 | 0.507 |
| **Depression** | NCCN | 83 | 0.003 | 1.313 | 0.998 | 1.003 | 0.076 | 13.163 | 0.368 | 0.507 |
| **Loss of interest**  **in everyday activities** | NCCN | 83 | 1.215 | 0.969 | 0.210 | 3.371 | 0.505 | 22.526 | 0.368 | 0.507 |
| **Concerns regading god** | NCCN | 79 | 21.608 | 40192,969 | 1.000 | 2423212264 | 0.001 | - | 0.027 | 0.037 |
| **Loss of faith** | NCCN | 79 | 0.247 | 0.946 | 0.794 | 1.280 | 0.201 | 8.168 | 0.027 | 0.037 |
| **Pain** | NCCN | 73 | -3.052 | 1.866 | 0.102 | 0.047 | 0.001 | 1.831 | 0.522 | 0.733 |
| **Nausea** | NCCN | 73 | 25.922 | 14126.428 | 0.999 | 1.810E+11 | 0.000 | - | 0.522 | 0.733 |
| **Fatigue** | NCCN | 73 | -3.266 | 1.712 | 0.056 | 0.038 | 0.001 | 1.094 | 0.522 | 0.733 |
| **Bathing/dressing** | NCCN | 73 | 2.694 | 2.378 | 0.257 | 14.795 | 0.140 | 1563.521 | 0.522 | 0.733 |
| **Physical appearance** | NCCN | 73 | 3.527 | 3.018 | 0.243 | 34.006 | 0.092 | 12607.828 | 0.522 | 0.733 |
| **Breathing** | NCCN | 73 | -1.088 | 2.108 | 0.606 | 0.337 | 0.005 | 20.979 | 0.522 | 0.733 |
| **Mouth sores** | NCCN | 73 | 2.116 | 2.197 | 0.336 | 8.296 | 0.112 | 615.650 | 0.522 | 0.733 |
| **Mouth dry** | NCCN | 73 | 2.483 | 1.907 | 0.193 | 11.981 | 0.285 | 503.183 | 0.522 | 0.733 |
| **Eating/Nutrition** | NCCN | 73 | -1.589 | 2.675 | 0.552 | 0.204 | 0.001 | 38.632 | 0.522 | 0.733 |
| **Indigestion** | NCCN | 73 | 1.090 | 2.095 | 0.603 | 2.974 | 0.049 | 180.597 | 0.522 | 0.733 |
| **Constipation** | NCCN | 73 | -1.919 | 2.384 | 0.421 | 0.174 | 0.001 | 15.697 | 0.522 | 0.733 |
| **Diarrhea** | NCCN | 73 | 0.489 | 1.951 | 0.802 | 1.630 | 0.036 | 74.679 | 0.522 | 0.733 |
| **Changes in urination** | NCCN | 73 | -0.892 | 2.470 | 0.718 | 0.410 | 0.003 | 51.935 | 0.522 | 0.733 |
| **Fevers** | NCCN | 73 | 1.568 | 3.118 | 0.615 | 4.799 | 0,011 | 2165.587 | 0.522 | 0.733 |
| **Nose dry/congested** | NCCN | 73 | 0.735 | 1.464 | 0.616 | 2.085 | 0.118 | 36.755 | 0.522 | 0.733 |
| **Tingling in hands/feet** | NCCN | 73 | -3.854 | 2.135 | 0.071 | 0.021 | 0.001 | 1.391 | 0.522 | 0.733 |
| **Feeling swollen/edema** | NCCN | 73 | 1.254 | 1.817 | 0.490 | 3.505 | 0.099 | 123.512 | 0.522 | 0.733 |
| **Memory/Concentration** | NCCN | 73 | -0.108 | 1.511 | 0.943 | 0.898 | 0.046 | 17.362 | 0.522 | 0.733 |
| **Sexual problems** | NCCN | 73 | -6.559 | 3.838 | 0.087 | 0.001 | 0.001 | 2.621 | 0.522 | 0.733 |

**Online supplementary table 3 Non-significant factors associated with an increased burden in DT (cut-off ≥5)**

A binary logistic regression was performed in the groups of the NCCN problem list and the MSQ (melanoma specific questions) to identify factors significantly associated with an indicated increased burden in DT or the need for psycho-oncological support (cut-off ≥5). If the above model does not specify an upper limit for the CI, the upper limit of the CI is undefined or infinite. This absence of an upper limit for the CI indicates that the model has an extremely high level of uncertainty in estimating the odds ratio, which significantly limits the interpretation and reliability of the factor. The following abbreviations are used: NCCN (National Comprehensive Cancer Network problem list), MSQ (melanoma specific questions), CI (confidence interval), Exp(B) (exponentiation of B), Sig. (significance).
